# Endotoxemia-induced inflammation and the effect on the human brain

**DOI:** 10.1186/cc9001

**Published:** 2010-05-05

**Authors:** Mark van den Boogaard, Bart P Ramakers, Nens van Alfen, Sieberen P van der Werf, Wilhelmina F Fick, Cornelia W Hoedemaekers, Marcel M Verbeek, Lisette Schoonhoven, Johannes G van der Hoeven, Peter Pickkers

**Affiliations:** 1 Department of Intensive Care Medicine, Radboud University Nijmegen Medical Centre, P.O. box 9101, Nijmegen, 6500HB, the Netherlands; 2 Department of Neurology and Clinical Neurophysiology, Radboud University Nijmegen Medical Centre, P.O. box 9101, Nijmegen, 6500HB, the Netherlands; 3 Department of Medical Psychology, Radboud University Nijmegen Medical Centre, P.O. box 9101, Nijmegen, 6500HB, the Netherlands; 4 Department of Neurology, Laboratory of Paediatrics and Neurology, Radboud University Nijmegen Medical Centre, P.O. box 9101, Nijmegen, 6500HB, the Netherlands; 5 Donders Institute for Brain, cognition and behaviour, Radboud University Nijmegen Medical Centre P.O. box 9101, Nijmegen, 6500HB, the Netherlands; 6 Department for IQ healthcare, Radboud University Nijmegen Medical Centre, P.O. box 9101, Nijmegen, 6500HB, the Netherlands

## Abstract

**Introduction:**

Effects of systemic inflammation on cerebral function are not clear, as both inflammation-induced encephalopathy as well as stress-hormone mediated alertness have been described.

**Methods:**

Experimental endotoxemia (2 ng/kg *Escherichia coli *lipopolysaccharide [LPS]) was induced in 15 subjects, whereas 10 served as controls. Cytokines (TNF-α, IL-6, IL1-RA and IL-10), cortisol, brain specific proteins (BSP), electroencephalography (EEG) and cognitive function tests (CFTs) were determined.

**Results:**

Following LPS infusion, circulating pro- and anti-inflammatory cytokines, and cortisol increased (*P *< 0.0001). BSP changes stayed within the normal range, in which neuron specific enolase (NSE) and S100-β changed significantly. Except in one subject with a mild encephalopathic episode, without cognitive dysfunction, endotoxemia induced no clinically relevant EEG changes. Quantitative EEG analysis showed a higher state of alertness detected by changes in the central region, and peak frequency in the occipital region. Improved CFTs during endotoxemia was found to be due to a practice effect as CFTs improved to the same extent in the reference group. Cortisol significantly correlated with a higher state of alertness detected on the EEG. Increased IL-10 and the decreased NSE both correlated with improvement of working memory and with psychomotor speed capacity. No other significant correlations between cytokines, cortisol, EEG, CFT and BSP were found.

**Conclusions:**

Short-term systemic inflammation does not provoke or explain the occurrence of septic encephalopathy, but primarily results in an inflammation-mediated increase in cortisol and alertness.

**Trial registration:**

NCT00513110.

## Introduction

With recorded prevalence rates of up to 70% [1], most patients with sepsis develop reversible brain dysfunction called sepsis-associated delirium or septic encephalopathy [2]. In patients suffering from septic encephalopathy, electroencephalographic (EEG) abnormalities have been observed [2], although there are conflicting results concerning elevated levels of serum brain specific proteins (BSP) in septic patients [3,4]. The mechanisms for brain dysfunction in septic patients are far from clear. Accumulating data suggest that circulating cytokines are associated with a neurotoxic effect in humans [1,2,5,6], either through a direct effect [7] or mediated via oxidative stress [8,9]. In addition, genetic variation in the IL-1β-converting enzyme resulting in chronically higher levels of IL-1β is associated with memory and learning deficits [10]. Moreover, there is evidence that increased levels of TNF-α and IL1-β further exacerbate ischemic and excitotoxic brain damage in humans [11,12].

On the other hand systemic inflammation induces a stress hormone response. This may lead to improvement of alertness, as throughout daytime temporal coupling between endogenous cortisol release and central alertness has been demonstrated in humans [13]. Also, elevated cortisol concentrations and cortisol administration [13-19] were shown to improve cognitive functions (CF). Intravenous administration of *Escherichia coli *lipopolysaccharide (LPS) to young healthy volunteers induces an acute systemic inflammatory response mediated by high levels of cytokines, resulting in oxidative stress [9,20,21] and increased levels of cortisol [22]. These effects are dose-dependent [23], and currently the administration of 2 or 4 ng/kg of LPS is mostly used in cases of experimental human endotoxemia. Human experimental endotoxemia can be used as a model to study the pathophysiological changes observed in septic patients, resulting in for example cardiac [24], vascular and endothelial dysfunction [21,25], coagulation abnormalities [26,27] and other subclinical end-organ dysfunction [28]. However, up to now the effects of experimental human endotoxemia on brain function has not been adequately investigated. Although high-dose LPS infusion in mice results in encephalopathy [29], experiments in humans demonstrated conflicting results. Experimental endotoxemia resulted in no change [30], deterioration [31] or improvement and deterioration of different cognitive function tests (CFTs) [22]. Endotoxemia-induced effects on EEG and BSP have not been investigated.

The aim of our present study was to investigate the effects of endotoxemia-induced inflammation on the brain. We addressed the question of whether LPS infusion induces changes in EEG, cortisol, BSPs, and CFs. Furthermore we wanted to examine if there is a correlation between the LPS-induced increased level of cytokines, cortisol, changes in EEG signals, BSPs and various CFs.

## Materials and methods

### Study design of human endotoxemia experiments

This study is registered at the Clinical Trial Register under the number NCT00513110. After approval of our ethics committee, 15 healthy male volunteers gave written informed consent to participate in the LPS study. Screening before the experiment revealed no abnormalities in medical history or physical examination. Routine laboratory tests and electrocardiogram (ECG) were normal and the volunteers had no reported brain dysfunction or psychiatric disorders. Ten healthy male volunteers were recruited for only cognitive measurements after they gave informed written consent.

During the experiment all 15 volunteers were monitored for heart rate (ECG), blood pressure (intra-arterially), body temperature (infrared tympanic thermometer; Sherwood Medical, 's-Hertogenbosch, the Netherlands) and EEG activity (Nicolet One system, Viasys Healthcare, Houten, The Netherlands), from about two hours before the administration of LPS and continued until the end of the experiment (about eight hours after the LPS administration). A cannula was inserted in a deep forearm vein for prehydration (1.5 L of 2.5% glucose/0.45 saline solution in the hour before LPS administration). During the first six hours after the LPS administration all subjects received 150 mL/h, and after that period until the end of the experiment 75 mL/h of 2.5% glucose/0.45 saline solution to ensure an optimal hydration status [32].

In one minute *E. coli *LPS 2 ng/kg was injected at t = 0 hours. The course of symptoms (headache, nausea, shivering, muscle pain and back pain) were scored on a six-point Likert scale; 0 = no symptoms, 5 = very severe symptoms, resulting in a total score of 0 to 25.

### Laboratory tests (cytokines, cortisol and brain specific proteins)

#### Analysis of cytokines and cortisol

All blood was allowed to clot and after centrifugation serum was stored at -80°C until analysis.

To determine the time course and peak values per individual, serial blood samples were taken. Cytokines concentrations of TNF-α, IL-6, IL-1-receptor antagonist, and IL-10 were measured in samples taken at baseline (t = 0) and at one, two, four and eight hours after LPS administration and batchwise analysed using Luminex assay. Cortisol levels were determined with luminometric immunoassay on a random access analyzer (Architect^® ^*i *System, Abbott, Illinois, USA) at baseline (t = 0) and at two, four and eight hours after LPS administration.

#### Analysis of brain specific proteins: S100-β, NSE, and GFAP

Proteins S100 calcium binding protein-β (S100-β) and neurospecific enolase (NSE) were analyzed using a commercially available monoclonal two-site luminometric assay (Sangtec Medical, Dietzenbach, Germany) according to the manufacturer's instructions using a Liaison automated analyzer (Byk Sangtec, Dietzenbach, Germany). The lower detection limit for S100-β is 0.02 μg/L. The upper reference range (95%) of S100-β serum concentrations in healthy subjects is 0.12 μg/L. The lower detection limit for NSE is 0.04 μg/L, and the upper reference range (95%) of NSE in serum from healthy subjects is 12.5 μg/L. The glial fibrillary acidic protein (GFAP) assay is a two-site luminometric assay. The serum sample is pipetted into coated wells of a microtitre strip containing the tracer antibody labelled with an isoluminol derivative. After incubation, the strips are washed and the chemiluminescent signal is measured in a luminometer. All steps of the assay are performed at room temperature. The lower detection limit for GFAP is 0.02 μg/L, and the upper limit (95%) of GFAP in serum in 75 healthy subjects was 0.49 μg/L.

### Electroencephalography

Subjects were monitored continuously with EEG, using a standard 21-lead recording with surface Ag/AgCl cup electrodes that were attached with Elefix EEG paste (Nihon Koden Inc., Foothill Ranch, California, USA) and placed according to the international 10-20 system. Recordings were made from electrode positions Fp1, Fp2, Fz, F3, F4, F7, F8, Cz, C3, C4, Pz, P3, P4, T3, T4, T5, T6, A1, A2, O1, and O2. Additional electrodes were placed for the recording of ocular movements and the ECG. Electrode impedance was kept below 5 KOhm, and the signals were filtered with a 1 Hz (high-pass) and 70 Hz (low-pass) filter. EEG signals were digitally sampled with a frequency of 256 Hz and stored on a computer hard disk. The full-length recordings were analyzed visually by an experienced clinical neurophysiologist (NvA) blinded to the LPS protocol. Raw EEGs were scored using a five category classification system for septic encephalopathies [33]. At least once per hour a one-minute artefact-free raw EEG sample (10-second epoch) of the subject lying awake with his eyes closed was selected for further quantitative analysis. In each subject, the power spectrum of samples was calculated for the standard frequency bands (delta <4 Hz; theta 4 to <8 Hz; alpha 8 to <13 Hz, beta >13 Hz) using Fourier transformation. The peak frequency in the occipital regions (P3 to O1 and P4 to O2 bipolar montages) was assessed for each time point. To detect changes in central alertness alpha and beta activity changes in the relative band power and absolute band power of the occipital and central electrodes (P4O2, P3O1 and F4C4, F3C3, respectively) were used, and also changes in peak frequency in the occipital region [13]. Changes in activity were expressed as percentage of change of the individual baseline level of activity before the LPS administration.

### Cognitive function tests

The anxiety level of each individual was measured at baseline after arrival at our research unit, with the Dutch State-Trait Anxiety Inventory (STAI) scale [34]. Higher scores (range 0 to 80) indicate higher levels of psychological distress. The time the participants required to finish the Grooved Pegboard test with the dominant hand served as an indication of fine motor control [35]. Working memory was assessed with the digit span forward and backward subtests of the Dutch translations of the Wechsler Adult Intelligence Scale (WAIS) III [36]. The total number of correct responses on the two-second stimulus interval condition of the Paced Auditory Serial Addition Test (PASAT) served as a measure for divided attention under time pressure [37]. The total number of correct responses on the Digit Symbol Test (SDT) of the WAIS III was chosen as an indication of psychomotor speed capacity as well as the information processing ability [36]. Reading speed, colour naming speed and distractibility were measured with the Stroop colour-word naming test [38] (Pearson Assessment and Inofrmation BV, Amsterdam, The Netherlands). To measure a possible practice effect as a result of test-retesting of the CFTs, the same CFTs under the same conditions and time intervals were performed in a reference group of 10 healthy male volunteers that did not receive LPS.

### Data analysis and statistics

All data were analyzed using SPSS version 16.01 (SPSS, Chicago, Illinois, USA). Results are expressed by means ± standard error of the mean or median (interquartile range (IQR)) depending on their distribution. LPS-induced effects were tested for significance with Friedman's analysis of variance (non-parametric test). To detect practice effect we compared the experimental group and the reference group with the repeated measurement-analysis of variance. Correlation analysis was performed with the Spearman's correlation coefficient. Because of the exploratory nature of this study, a correction for multiple testing was not included. Statistical significance was defined as a *P *value less than 0.05.

## Results

### Baseline characteristics

Baseline characteristics of the 15 healthy male volunteers are shown in Table 1. All participants had a mean age of 23 ± 2 years, and had a high (college or university) educational level.

**Table 1:** Baseline demographic characteristics of the study group

Characteristic (n = 15)	
Age (years)	23 ± 2
Height (cm)	186 ± 7
Weight (kg)	77.1 ± 9.0
Body mass index (kg/m^2^)	22.3 ± 2.0
Systolic blood pressure (mmHg)	130 ± 6
Diastolic blood pressure (mmHg)	65 ± 9
Heart rate (bpm)	61 ± 8
Temperature (°C)	35.7 ± 0.3
Symptom score (median)	0 (interquartile range 0-1)

All values are means ± standard deviation unless other reported.

### LPS-induced changes in clinical and inflammatory parameters and cortisol levels

LPS administration induced the expected transient flu-like symptoms. Body temperature increased by 1.4 ± 0.1°C (*P *< 0.0001) and heart rate by 27 ± 2 bpm (*P *< 0.0001). Cumulative symptom scores increased from a median score of 0 (IQR 0 to 1) to 4 (IQR 2 to 7) at 70 minutes after LPS administration, after which there was a decrease to a median of 2 (IQR 1 to 5) and 1 (IQR 0 to 2) at two and four hours, respectively (*P *< 0.0001). Relevant to the present study, LPS administration induced an increase in headache score from 0 score to a maximum of 2 (IQR 1 to 3) at 90 minutes after endotoxin administration (*P *< 0.0001).

**Figure 1:**
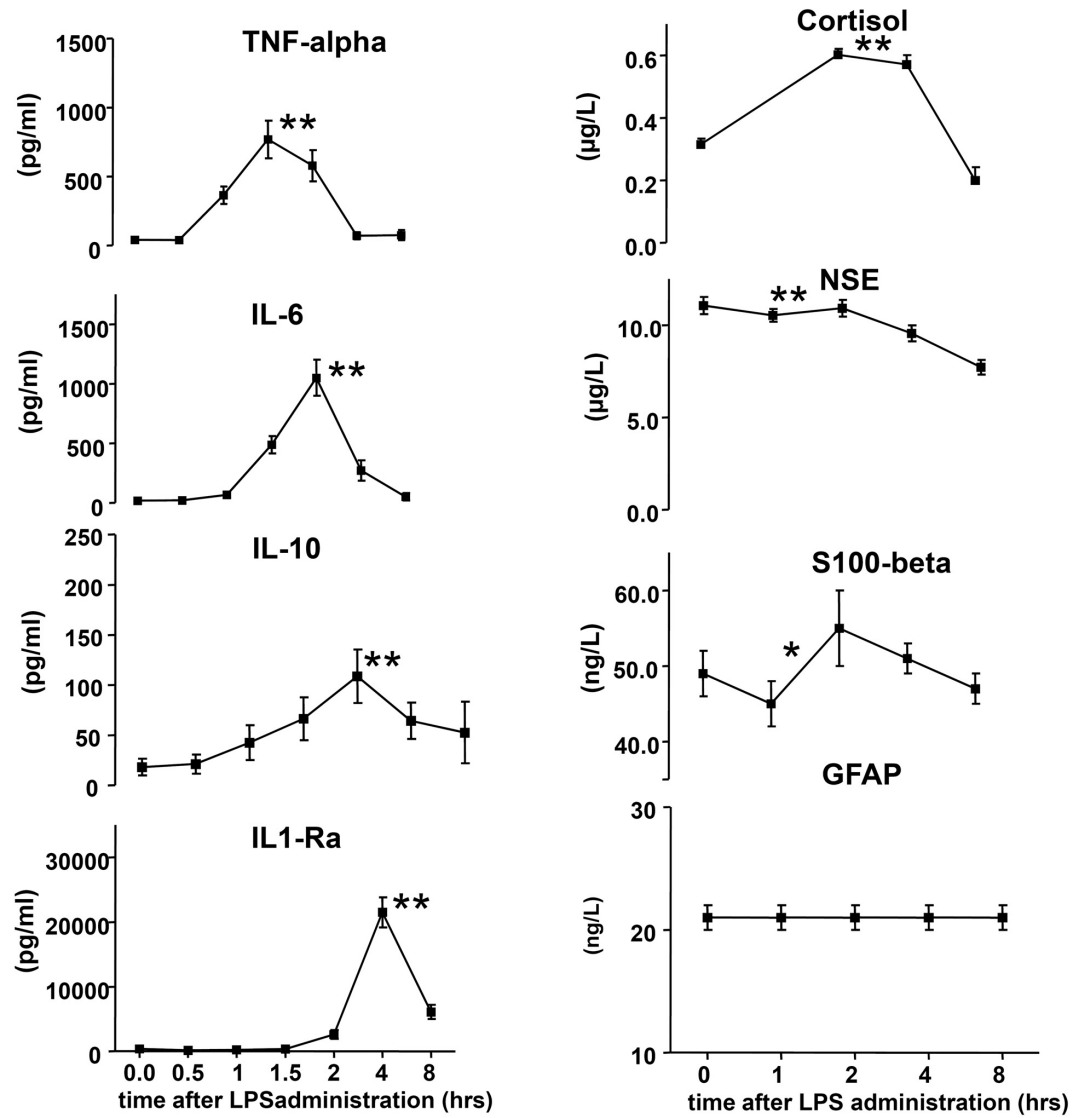
**LPS-induced changes in cytokine plasma concentrations, cortisol and brain specific proteins**. Time -0- reflects baseline concentrations. Administration of lipopolysaccharide (LPS) resulted in a marked increase in TNF-α, IL-6, IL-10, IL-1Ra and cortisol concentrations. All changes in cytokine and the cortisol concentrations were significant (*P *< 0.001). Concentrations of neuron specific enolase (NSE) decreased after administration of LPS (*P *< 0.001) and S100-β showed a significant biphasic change (*P *= 0.038). All data are expressed as mean ± standard error of the mean (n = 15). GFAP, glial fibrillary acidic protein; S100β, S100 Calcium Binding Protein B. * *P *< 0.05. ** *P *< 0.001.

All plasma cytokine concentrations increased significantly (all *P *< 0.0001) after the administration of LPS (Figure 1). Cortisol levels increased significantly from 0.31 ± 0.07 to 0.60 ± 0.07 μmol/l (*P *< 0.0001) two hours after LPS administration and dropped to baseline levels eight hours after LPS administration (Figure 1).

### LPS-induced changes in brain specific proteins

As illustrated in Figure 1, NSE levels showed a small, but statistically significant decrease from 11.1 ± 0.47 to 7.7 ± 0.39 μg/L after the administration of LPS (*P *< 0.0001). S100-β showed a significant biphasic change (from 0.049 ± 0.002 up to 0.055 ± 0.004 and down to 0.047 ± 0.002 μg/L, *P *= 0.04), whereas GFAP levels did not change significantly (*P *= 0.41).

### LPS-induced changes in EEG

#### Visual analysis

For each subject, at least eight hours of raw EEG were available for visual analysis. All EEGs before LPS infusion were within the normal range. One hour after LPS infusion mild transient encephalopathic EEG changes in the theta range were present in one subject for 15 minutes, without associated cognitive impairment. Of note, this subject had a very low cytokine response during endotoxemia (TNF-α level of 169 pg/ml compared with the group mean of 814 ± 133 pg/ml, and IL-6 level of 508 pg/ml compared with the group mean of 1,111 ± 142 pg/ml) and an average cortisol response (0.29 to 0.67 μmol/l). The EEGs from the other 14 subjects remained within the normal range after LPS infusion, and no focal or epileptiform abnormalities were found.

#### Quantitative analysis

**Figure 2:**
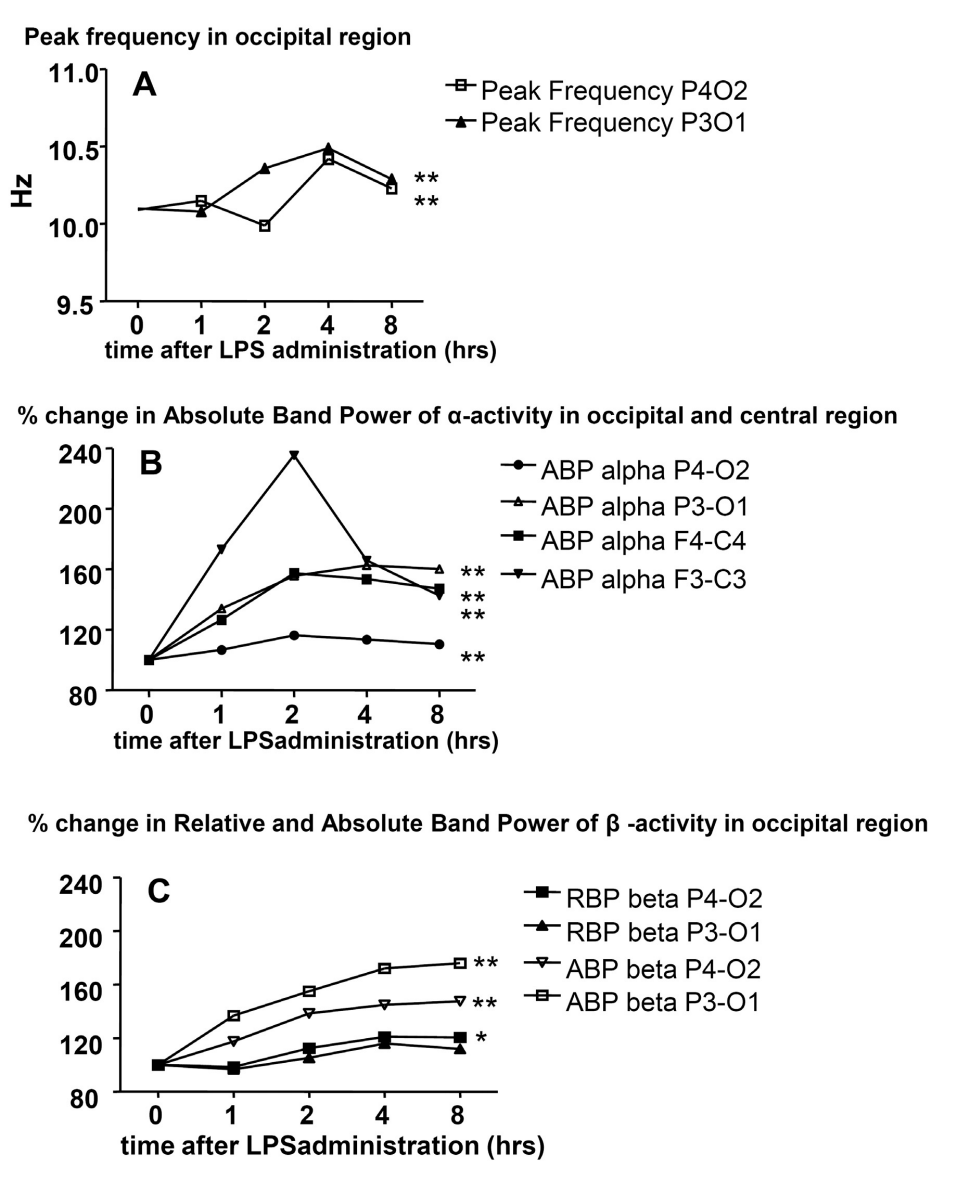
**Increase of the EEG occipital peak frequencies, relative alpha band power and absolute alpha and beta band power two to three hours after LPS infusion**. Data of peak frequency are absolute numbers, data of absolute and relative band power are expressed as percentage changes. Time -0- reflects baseline measurements. (standard error of the means were omitted for reasons of clarity). * *P *< 0.05. ** *P *< 0.001. **(a) **Peak frequency in occipital region. Friedman analysis of variance revealed changes in P4O2 and P3O1 (both *P *< 0.001). **(b) **Percentage change compared to baseline in absolute band power (ABP) of alpha activity in occipital and central region. Friedman analysis of variance revealed changes for alpha activity in P4O, P3O1 and F4C4, F3C3 all *P *< 0.001. **(c) **Percentage change compared with baseline in absolute band power (ABP) and relative band power (RBP) of beta activity in occipital region. Friedman analysis of variance revealed changes of RBP for beta activity in P4O2 (*P *= 0.017), P3O1 (*P *= 0.575) and ABP for beta activity in P4O and P3O1 (both *P *< 0.001).

LPS induced a significant increase of the peak frequency and absolute band power of alpha and beta activity in the occipital region, P4O2 and P3O1 (all *P *< 0.0001). The absolute power of the alpha activity in the central region, F4C4 and F3C3, increased significantly (both *P *< 0.0001). The relative band power of the beta activity in P4O2 increased significantly (*P *= 0.017), indicating a higher state of alertness. No other relevant EEG changes were found (Figure 2).

### LPS-induced changes in cognitive function

Baseline STAI in the LPS group was 32.7 ± 1.5, indicating a low level of anxiety that did not differ from the reference group 29.1 ± 3.7 (*P *= 0.13). During endotoxemia all measured CFs significantly improved. These improvements were not significantly different from those observed in the reference group who did not receive LPS (Table 2), indicating that the improvement of the CFT in the LPS group was due to a practice effect.

**Table 2:** Neuropsychological test outcomes (mean ± SD) at 0 (baseline), 2 and 8 hours after LPS administration

	LPS group (n = 15)				Reference group (n = 10)				*P* value (between group)
			
Age (Dutch) STAI total	*22.8 *± 2.2				*25.5 *± 2.5				0.87*
			
	32.7 ± 1.5				29.1 ± 3.7				0.13*
			
Neuropsychological test	t = 0	t = 2	t = 8	*P* value (within group)	t = 0	t = 2	t = 8	*P* value (within group)	
Stroop A (in seconds) ^1^	39 ± 2	35 ± 2	35 ± 2	*0.0001*	37 ± 5	34 ± 4	34 ± 4	*0.001*	*0.49*
Stroop B (in seconds) ^1^	51 ± 3	45 ± 3	43 ± 2	*0.0001*	48 ± 7	44 ± 7	43 ± 7	*0.001*	*0.45*
Stroop C (in seconds) ^1^	75 ± 6	65 ± 4	64 ± 4	*0.003*	67 ± 10	62 ± 12	61 ± 11	*0.004*	*0.23*
Pasat ^2^	49 ± 2	50 ± 2	56 ± 2	*0.001*	50 ± 7	54 ± 4	54 ± 5	*0.031*	*0.07*
Digits forward ^2^	11 ± 1	12 ± 1	11 ± 1	*0.115*	10 ± 2	11 ± 1	11 ± 2	*0.235*	*0.81*
Digits backward ^2^	8 ± 1	9 ± 1	9 ± 1	*0.30*	9 ± 2	9 ± 1	9 ± 2	*0.454*	*0.65*
Digits total ^2^	19 ± 1	20 ± 1	20 ± 1	*0.066*	19 ± 4	20 ± 3	21 ± 4	*0.203*	*0.63*
Pegboard ^1^	64 ± 2	59 ± 2	61 ± 2	*0.037*	58 ± 5	56 ± 6	56 ± 7	*0.362*	*0.35*
Symbol substitution task^2^	87 ± 3	99 ± 4	101 ± 3	*0.0001*	98 ± 14	108 ± 17	112 ± 19	*0.0001*	*0.53*

All values are means ± SD unless other reported.

* Unpaired T-test.

^1 ^Decrease indicates an improvement of the test.

^2 ^Increase indicates an improvement of the test.

Reading speed was measured by Stroop A-B-C word naming test.

Attention under time pressure was measured by the paced auditory serial addition test (PASAT).

Working memory was tested in numbers with the Digits forward and backward test.

The fine motor control was tested with the Grooved Pegboard test.

Psychomotor speed capacity was measured by the symbol substitution task.

LPS, lipopolysaccharide; SD, standard deviation; STAI, Dutch State-Trait Anxiety Inventory scale.

### Correlation analyses

#### Cytokines, cortisol, BSP, EEG, and CF

To analyse the effects between the measured cytokine levels, cortisol, BSP levels, EEG parameters and cognitive performances, data were correlated.

In the LPS group the elevated levels of the anti-inflammatory cytokine IL-10 significantly correlated with the improvement of the working memory (*r *= 0.71, *P *= 0.003) and the psychomotor speed capacity (*r *= 0.71, *P *= 0.003). The increased cortisol levels significantly correlated with the increased peak frequency in the occipital electrodes P4O2 (r = 0.61, *P *= 0.016) and P3O1 (*r *= 0.69, *P *= 0.005). In the LPS group, the decreased level of NSE significantly correlated with the improvement of the working memory and psychomotor speed capacity (*r *= -0.53, *P *= 0.048 and *r *= -0.67, *P *= 0.006, respectively). The increased alpha activity in F3C3 central region correlated significantly with the improvement of the working memory (*r *= 0.66, *P *= 0.007). No other correlations between cytokines, cortisol, BSP, EEG and CF were found.

## Discussion

The main result of the present study is that, despite very high cytokine concentrations during experimental endotoxemia, no indications were found that acute systemic inflammation results in increased levels of BSPs and deterioration of CFs in humans *in vivo*. In addition, a group level quantitative EEG analysis showed a higher state of alertness that correlated with cortisol concentrations. Nevertheless, the concomitant improvement in CFTs turned out to represent a practice effect as a similar improvement was observed in subjects who did not receive LPS. Although the increased alpha activity in the central region of the brain correlated with the improvement of working memory in the LPS group, it appears conceivable that this correlation may also be present in the control group during the repeated CFTs, but this finding needs to be confirmed. Interestingly, the one subject with a transient mild encephalopathic episode on EEG, that is category 2 following the score used by Young and colleagues [33], showed that this was not associated with objective cognitive dysfunction. In addition, this subject had one of the lowest LPS-induced proinflammatory cytokine responses of the whole group, arguing against a cytokine-mediated effect.

Although experimental endotoxemia in young humans without any co-morbidity mimics the pathophysiological changes in septic patients in many ways, important differences also exist. For example, TNF-α concentrations found during experimental endotoxemia are much higher than in septic patients, whereas other cytokines are released to a lesser extent and some inflammatory mediators found in septic patients are not induced during experimental endotoxemia [39]. It appears likely that the relatively mild insult and short duration of elevated cytokine levels during experimental endotoxemia does account for the increase in cortisol concentration and observed stimulating effects on the brain, but may not reflect the neurotoxic effects of inflammatory mediators present in septic patients. In addition, age and the pre-existing neurological situation is likely to be important. Healthy elderly people show a more pronounced inflammatory response during experimental endotoxemia [40] and pre-existing micro-glial inflammation primes the brain for development of cognitive impairment in non-infectious and infectious central nervous system dysfunction [41]. Therefore, although our study shows that a short duration of very high cytokine levels is not associated with brain dysfunction it does not exclude the possible effects of cytokines on neurons in older ICU patients with co-morbidities.

Cortisol secretion is related to electroencephalographic alertness [13]. We showed a significant correlation between the elevated levels of cortisol and the change in occipital peak frequency. It is likely that this higher state of alertness was due to the LPS-induced inflammation with feelings of sickness resulting in a stress hormone-driven 'flight-fight' response [42], which is also associated with increased cortisol. This appears to be a short-lived effect, because chronically elevated levels of glucocorticoids result in a deterioration of CF [43]. As a result of this, it is possible that in the septic patient the stimulating effect of stress hormones on the brain is overshadowed by the neurotoxic effect of persistently elevated level of cytokines and other mediators. In septic patients, levels of some proinflammatory cytokines are not as high as in the LPS model, but the duration of the elevated cytokine level is much longer [44]. If these cytokines play a role in the sepsis-associated encephalopathy, it is apparently not the absolute peak concentration of the proinflammatory cytokine that is of importance. Presumably, sustained elevated levels of cytokines are more important in the development of organ failure and brain dysfunction in sepsis. In accordance, chronic small increases in proinflammatory cytokine levels due to polymorphisms were found to be associated with decreased brain function [10]. Naturally, other not yet identified mediators of inflammation that may be increased in septic patients but not during experimental endotoxemia may also account for brain dysfunction observed in septic patients.

In previous studies with much lower doses of LPS (0.2 to 0.8 ng/kg), with little systemic inflammatory response, conflicting effects on CFs were reported [22,30,31]. Compared with experiments with 0.2 ng/kg, improvement of working memory was shown in a study with 10 healthy volunteers with a dose of 0.8 ng/kg LPS [22]. In these studies, cortisol level and cytokines increased slightly, compared with our results [22,30,31], which is associated with dysfunction of other organs [24,28,45]. Furthermore, a potential problem in the studies with low doses of LPS was that no correction for practice effect was performed while practice effects during CFT are common, especially in situations with short test-retest intervals. Our study demonstrates that the observed improvement in CFs after LPS infusion in all domains was due to a practice effect. Without the use of a control group and the measurement of practice effect results are bound to be misinterpreted. Our results suggest that a short-term inflammation does not influence practice effect or lead to a significant deterioration or improvement of CFs.

The observed relations between EEG changes and inflammatory markers indicate a higher state of inflammation-induced alertness. Higher dosages of LPS result in higher levels of cytokines [23] and more elevated levels of cortisol result in a higher state of alertness [13]. The higher state of alertness during endotoxemia is possibly a so-called fight and flight response, rather than being due to the increased cytokine concentrations.

Although it is tempting to speculate, due to the observational nature of the present study we cannot conclude whether or not the anti-inflammatory innate immune response, measured by IL-10, exerts a protective effect on the brain, and this correlation needs further study. In addition, the pathophysiological mechanism by which systemic inflammation results in the observed decrease of NSE is not clear. Increased levels of NSE are associated with deterioration of CF after cardiac surgery [46]. Also, increased NSE levels are associated with brain injury in septic patients, but an association between NSE and CFs in septic patients has not been examined.

## Conclusions

Administration of LPS to humans results in systemic inflammation with high levels of cytokines and increased cortisol levels. In young healthy volunteers this can sporadically lead to a transient mild deterioration of brain function without clinical correlation. Overall, LPS infusion results in a higher state of alertness determined on the EEG, while the practice effects in CFTs are not significantly influenced. Short-term systemic inflammation does not provoke or explain the occurrence of a septic encephalopathy.

## Key messages

• Despite very high cytokine concentrations during experimental endotoxemia, no indications were found that acute systemic inflammation results in increases of BSPs and deterioration of CFs in humans *in vivo*.

• LPS-induced increases in cortisol significantly correlated with a higher state of alertness detected on the EEG.

• Although most of the improvements in CF were identified as practice effects, increased IL-10 and the decreased NSE both correlated with improvement of working memory and with psychomotor speed capacity.

• An acute systemic inflammation induced by LPS does not suppress this practice effect in CFTs.

## Abbreviations

BSP: brain specific proteins; CF: cognitive function; CFT: cognitive function tests; ECG: electrocardiogram; EEG: electroencephalography; GFAP: glial fibrillary acidic protein; IL: interleukin; IQR: interquartile range; LPS: lipopolysaccharide; NSE: neurospecific enolase; PASAT: paced auditory serial addition test; S100-β: S100 calcium binding protein-β; SDT: digit symbol test; STAI: state-trait anxiety inventory; TNF-α: tumor necrosis factor-α; WAIS-III: wechsler adult intelligence scale III.

## Competing interests

The authors declare that they have no competing interests.

## Authors' contributions

MvdB and BR carried out the study, gathered all data and, with WF, performed the statistical analysis. NvA performed the EEG analysis. SvdW performed the CFT analysis. MV performed the BSP blood analysis. PP, LS and CH supervised the conduct of the study and writing of the paper. JvdH corrected the manuscript. All authors read and approved the final manuscript.
